# Computational analysis of functional single nucleotide polymorphisms associated with SLC26A4 gene

**DOI:** 10.1371/journal.pone.0225368

**Published:** 2020-01-23

**Authors:** Mirza Jawad Ul Hasnain, Muhammad Shoaib, Salman Qadri, Bakhtawar Afzal, Tehreem Anwar, Syed Hassan Abbas, Amina Sarwar, Hafiz Muhammad Talha Malik, Muhammad Tariq Pervez

**Affiliations:** 1 Department of Bioinformatics, Virtual University of Pakistan, Lahore, Pakistan; 2 Department of Computer Science and Engineering, UET, Lahore, Pakistan; 3 Department of CS & IT, The Islamia University of Bahawalpur, Bahawalpur, Pakistan; 4 Department of Biosciences, COMSATS University, Islamabad, Pakistan; 5 Alpha Genomics Private Limited, PWD, Islamabad, Pakistan; King Abdulaziz University, SAUDI ARABIA

## Abstract

Single Nucleotide Polymorphisms (SNPs) are the most common candidate mutations in human beings that play a vital role in the genetic basis of certain diseases. Previous studies revealed that Solute Carrier Family 26 Member 4 (SLC26A4) being an essential gene of the multi-faceted transporter family SLC26 facilitates reflexive movement of Iodide into follicular lumen through apical membrane of thyrocyte. SLC26A4 gene encodes Pendred protein, a membrane glycoprotein, highly hydrophobic in nature, present at the apical membrane of thyrocyte functioning as transporter of iodide for thyroid cells. A minor genetic variation in SLC26A4 can cause Pendred syndrome, a syndrome associated with thyroid glands and deafness. In this study, we performed *in-silico* analysis of 674 missense SNPs of SLC26A4 using different computational platforms. The bunch of tools including SNPNEXUS, SNAP-2, PhD-SNP, SNPs&GO, I-Mutant, ConSurf, and ModPred were used to predict 23 highly confident damaging and disease causing nsSNPs (G209V, G197R, L458P, S427P, Q101P, W472R, N392Y, V359E, R409C, Q235R, R409P, G139V, G497S, H723R, D87G, Y127H, F667C, G334A, G95R, S427C, R291W, Q383H and E384G) that could potentially alter the SLC26A4 gene. Moreover, protein structure prediction, protein-ligand docking and Molecular Dynamics simulation were performed to confirm the impact of two evident alterations (Y127H and G334A) on the protein structure and function.

## Introduction

In last few years, genome-wide association studies (GWAS) tested a huge number of SNPs in thousands of people and highlighted reproducibly distinguished numerous relationships among the common genetic variants and diseases with their traits [1]. These studies have advanced from measuring 100,000 SNPs to one million, and test sizes have increased significantly as the need of variations that make the analysis of diseases easier has escalated [2]. The fast increment in the quantity of GWAS has given a phenomenal chance to look at the potential effect on the complex diseases of the common genetic variants by methodically recording and condensing key attributes of the inferred associations and their associated SNPs respectively [3].

Single nucleotide polymorphisms (SNPs) act as indicators in the association and linkage studies for detecting the part of genome involved in a particular disease [4]. The polymorphisms present in the coding and the regulatory regions may themselves be embroiled in the diseases [5]. A SNP that causes an amino acid substitution is termed as Non-Synonymous SNP and is of great focus and interest due to the huge number of amino acid variations that are known to lead towards the gene lesions that cause diseases [6]. The studies to detect SNPs along with the mutagenesis analysis complement each other to identify the amino acid substitutions in the protein coding regions, as each variation can potentially alter the function or structure of a protein [7].

In the present world of genetics, a major goal remains to comprehend the substantial part of the disease-causing genetic mutations and variants [8]. Characterizing the variants on the basis of their nature, organizing an extensive study on analysis of SNPs associated with a gene relating to a particular disease and carrying out in depth associative studies are a requirement of the current era [9]. Until now, our understanding of human gene mutations and variations remains elementary. According to Annotation Release 109, GRCh38.p12 of Homo sapiens, the cytogenic location of SLC26A4 is 7q22.3, that means it is located at long arm of chromosome known as q arm at position 222.3, whereas the gene is molecularly located at base pairs 107,660,635 to 107,717,809 on chromosome 7 [10].

The human population has moderately restricted genetic variations. Numerous uncommon hereditary variations exist in the humans, yet the majority of the heterozygosity in the populace is inferable from commonly existing alleles [11]. The rare variations incorporate the essential drivers of uncommon, Mendelian diseases, having these alleles commonly being later in root and exceptionally penetrant. Whereas, a few also believe that the basic variations may contribute fundamentally to hereditary hazard for the common diseases to occur [12]. The approach towards the common diseases caused by common variants leads to the Pendrin protein causing a set of abnormalities. SLC26A4 gene has some genetic variations that are identified to be involved in both non-syndromic deafness related with vestibular aqueduct enlargement and Pendred syndrome, and it is necessary to study molecular confirmation of Pendred Syndrome Gene in diagnosis of these diseases [13].

Pendrin protein produced by the translation of SLC26A4 gene, is a profoundly hydrophobic protein comprising of total 780 amino acids [14]. Along with 12 transmembrane domains it has amino-and carboxyterminus located in it [15]. In one of the intracellular carboxyterminus, pendrin protein comprises of a supposed sulfate transporter and a domain, antisigma factor opponent,STAS [16] which has known to be involved along with pendrin in communicating with cystic fibrosis conductance controller regulatory space (CFTR) in particular epithelia [17]. By communicating the PDS gene in heterologous cell frameworks, it was exhibited that pendrin was playing a role as a transporter of various chloride, anions, formate, iodide, and bicarbonate, however not sulfate, notwithstanding a nearby homology between this protein and most of the sulfate transporters [18].

The study of candidate genes tends to decrease the quantity of SNPs to a set of mutations from which the genes are destined to build up the genetic foundation of disease under consideration [14]. Although, if extensive data sets of the candidate genes are viewed as, different testing of hundreds or even thousand number of SNPs for our situation too makes the detection of association troublesome[19]. A conceivable method to conquer the issue of testing overpowering quantities of SNPs, particularly on account of the candidate gene studies, is to organize SNPs as per their priority of functional significance [12]. Prior natural information in the existing databases can be utilized to lessen the quantity of SNPs by concentrating on particular genomic regions, the computational methodologies and aptitude is utilized to segregate between the neutral SNPs and the SNPs of likely functional importance [20].

These days, more refined computational methods are bieng created, that help in developing high-throughput, practical path in identification of change in structure, capacity and strength of protein as result of some variation [7,21]. Keeping in view the essentialities, the current study was carried out to identify and predict pathogenic SNPs in *SLC26A4* gene, their disease associations and the effect of deleterious nsSNPs in the protein structural behavior. For the prediction of pathogenic nsSNPs in *SLC26A4* gene, we used SNPNEXUS [22], SNAP2 [23], SNPs&GO [24], PhD-SNP (Predictor of human deleterious single nucleotide polymorphisms) [25], I-Mutant [26] and Mu-Pro [27]. For identifying amino acid residues conservation ConSurf [28] was used. ModPred [29] was used to identify post translational modification sites in the protein. SPARK-X [30] provided platform for generating three dimensional structures of mutant and native PDS protein and these srtructures were refined by using ModRefiner [31]. In order to see the structural variance and difference in interacting behaviors of native and mutants we did protein-ligand docking where COACH was used for predicting ligand binding sites. PyMOL [32] was used for protein-ligand docking to observe the structural variation of native and mutant proteins with surrounding ligands. LigPlot was used for the visualization of three dimensional structures of protein and for identifying the interactive behavior of both native and mutated amino acids with ligands. For the clear depiction of structural variantions with time, Molecular Dynamics simulation was done by GROMACS 5.1.2 [33].

## Materials and methods

**Fig 1:**
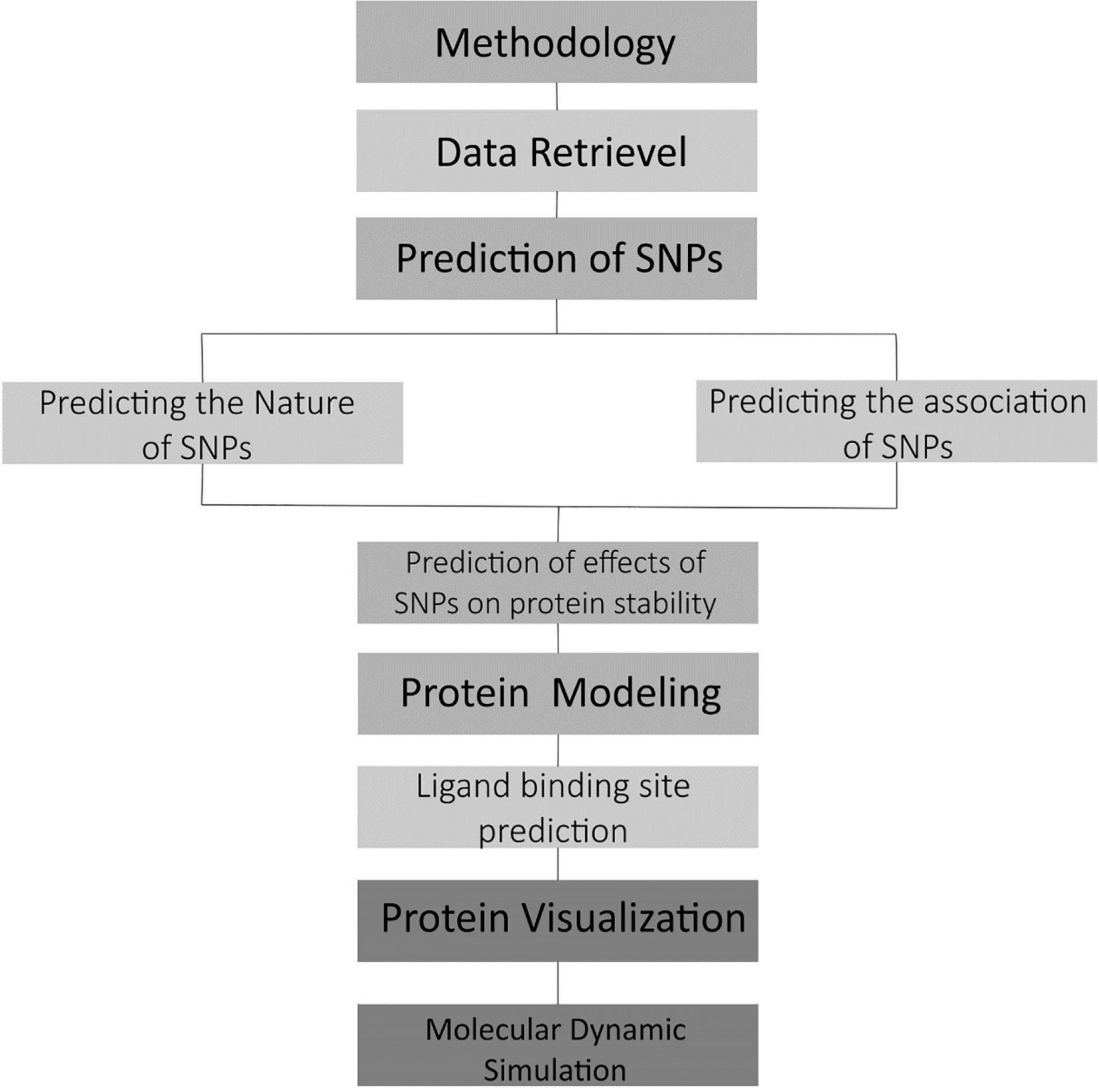
Schematic representation of the overall schema followed in the study.

The schematic illustration of the methodology utilized in this study is given in Fig 1.

### Dataset download

The nucleotide sequence in FASTA format (Accession number: NC_000007. 14), Pendrin protein amino acid sequence (NP_000432.1) were downloaded from NCBI (http://www.ncbi.nlm.nih.gov) and datasets related to SNPs of SLC26A4 gene were obtained from NCBI database of SNPs **(**dbSNP (http://www.ncbi.nlm.nih.gov/snp/). Whereas data regarding Pendrin gene and protein were obtained from OMIM database (http://www.omim.org).

### Prediction of functional impact of SNPs

The effects of SNPs were predicted using certain online tools and servers including SNPNEXUS (https://www.snp-nexus.org) and SNAP2 (https://www.rostlab.org/servces/snap/). server. Out of which SNPNEXUS has further embedded tools like SIFT and PolyPhen. Both of the servers take into account different inputs and classify the possible SNPs on the basis of their respective confidence scores. For non-synonymous single amino acid substitution, SNPNEXUS provides the predicted effect on protein function (Tolerated or Damaging) based on the SIFT and PolyPhen predictions [22]. SIFT classifies the variants based on a tolerance index score and those with score ≤0.05 are considered to be deleterious. Whereas, the PolyPhen output consists of a score that ranges from 0 to 1, with zero indicating a neutral effect of amino acid substitutions on protein function and 1 indicates the most damaging behavior. SNAP2 enables a comparison across the genomes while predicting functional effect of changes at amino acid level [23]. The common nsSNPs from both the servers were taken into account for further analysis.

### Prediction of disease association of SNPs

PhD-SNP (http://snps.biofold.org/phd-snp/phd-snp.html) and SNPS&GO (http://snps-and-go.biocomp.unibo.it/snps-and-go/) were used for determining association of filtered SNPs with disease. PhD-SNP is an online tool used to predict the association of diseases with the SNPs with an accuracy rate of 78%. Classifying the SNPs into disease associated or neutral it ranks them on a scale of 0 to 9 [24]. SNPs&GO is an accurate tool which predicts disease associated amino acid change at a single position in a specific protein including functional classifications with overall 82% prediction accuracy [25]. The inputs given to SNPs&GO were the UniProt accession number (043511) of Pendrin protein and mutation position of both native and mutated amino acid.

### Impact on protein stability

The SNPs tend to affect protein strength by either decreasing or increasing the protein stability. To predict these effects, a pair of tools was used to maximize the confidence of the alterations caused. I-Mutant (http://folding.biofold.org/i-mutant/i-mutant2.0.html), predicts the impact of SNPs in changing stable state of a protein. The accuracy of the tool goes up to 77% [26]. The input for I-Mutant was pendrin protein amino acid sequence and mutations of residues along with positions. MUpro, a group containing many machine learning programs, identifies the differences in protein states and strength due to the impact of amino acid mutation [27]. The inputs for MUpro was as that of I-Mutant but MUpro also takes positions of substitutions along with original and mutated residue.

### Sequence conservation analysis

ConSurf, an online tool was used for Pendrin protein conservation analysis (http://ConSurf.tau.ac.il/). ConSurf is an efficient tool for predicting the high-throughput functionalities of the target regions of the proteins. For every residue of the protein of interest, the conservation analysis is shown on a scale of 1 to 9. On the scale, 1–3 score is referred as variable, 4–6 as average and 7–9 scores are showing highly conserved regions [28]. The tool takes FASTA protein sequence as an input.

### Post transcriptional modifications sites (PTMs) prediction

The Post-transcriptional modification sites comprise of numerous kinds of amino acid modifications which give rise to the broad spectrum of proteins being generated. Methylation, phosphorylation, acetylation and ubiquitination are some of the characterized PTM sites. These sites play a vital role in important aspects of cellular organization like protein-protein interactions and signaling pathways that are associated with diseases. Thus, predicting PTM information helps understand the effect of variations in terms of disease association or pathogenicity. ModPred was used for predicting the overall PTMs (http://www.modpred.org/). ModPred by using protein sequences predicts PTMs within them. It incorporates 34 gatherings of strategic relapse abstractions that are prepared autonomously using a group of 126,036 non-excess practically confirmed sites for 23 distinct variations, accomplished by consulting open databases [29].

### Protein modelling

A structural analysis was performed to evaluate the structural stability of the native and mutant proteins. As the structure of PDS protein is not reported yet so we predicted the native protein structure using MODELLER 9.11 package. FOLDX was used for refinement, repairing, energy minimization and mutant generation using native predicted structure of VHL protein. For the verification of protein models SAVES server (http://servicesn.mbi.ucla.edu/SAVES/) was used. It has a set of six integrated modules including RAMACHANDRAN plot which provides information about conformation of residues in allowed and disallowed regions and is used for structural evaluation of protein [34].

### Prediction of binding sites for ligand & docking

Molecular docking analysis was performed to identify structural variations of native and wild type PDS protein structures by comparing the binding affinities of six ligands (4KU, BCT, DMU, IOD, MG, XAN) with these structures. In order to see the structural variations among native and mutant structures, we first predicted the binding sites for ligands in the Pendrin protein using COACH (http://zhanglab.ccmb.med.umich.edu/COACH/)which is a tool that uses binding site approaches or interactions prediction, particularly binding sites of proteins and ligands. COACH uses two comparative methods TM_SITE and SSITE for identification of binding sites for ligands [35]. PyMol was used for docking all three structures with ligands separately. The interacting residues of native and mutant protein structures with neighbor ligands were further analyzed by LigPlot and the resultant 2D depictions were compared to identify any possible changes in interacting residues caused by the mutation.

### Molecular dynamics simulations

In order to compare the structural changes with time, we performed Molecular Dynamics (MD) simulation of native and mutant structures using GROMACS version 5.1.2 that was executed on Z-book computing machine with 16 GB RAM and Ubuntu 18.04. Initial calculation was performed using GROMACS-OPLS-AA force field. Systems were solvated in a cubic box with water molecules at 1 nm (10 Å) marginal radius and electrical neutralize of systems was done by adding 10 sodium Na^+^ to the simulation box using genion tool in the GROMACS. Steepest descent minimization algorithm with energy step size 0.01 and maximum number of iteration 50000 was used for the energy minimization. Berendsen temperature (tcouple) of 300K and Parrinello-Rahman (pcouple) pressure of 1 bar were used to keep the system in stable environment whereas the coupling constant was adjusted at 0.1 and 2.0 ps for temperature and pressure respectively. Partial Mesh Ewald (PME) algorithm was used for all electrostatic interactions. Short-range cut-off for van der wall (rvdw) and electrostatic (rcoulomb) was fixed at 1.0 ns whereas; neighbor list (nstlist) was set at 10 ns. LINCS algorithm was used to constrain all bonds including heavy atom-H bonds and the time step was set at 0.002 ps. Isothermal compressibility was set at 4.5x10^-5^. Structures were equilibrated for 100 ps in NPT (pcoupl) and NVT (tcouple). Lastly, 10-ns MDS was performed for both native and mutant structures and trajectories were stored every 1 ps. We then computed the comparative analysis of structural deviations among wild type and mutant structures. RMSD, RMSF, SASA, Rg, temperature, pressure and density plot analysis were carried out using g_rms, g_rmsf, g_sasa, g_Rg and g_density tools, respectively. All plots were generated using XMGRACE program.

## Results

**Fig 2:**
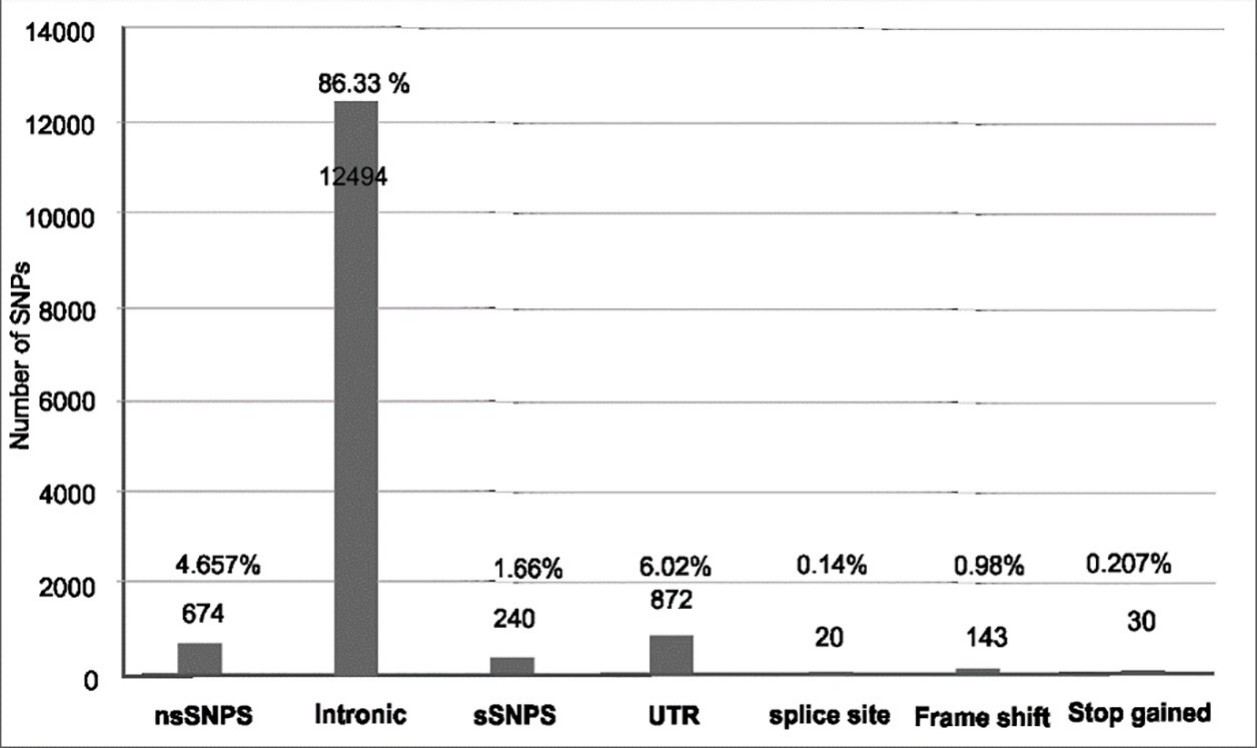
Distribution of SNPs.

Human SLC2A46 gene contains a total number of 14473 single nucleotide polymorphisms). Out of which 674 were non-synonymous SNPs (4.70%), 12494 lay in intronic regions (86.30%), 240 were reported to be synonymous SNPs (1.80%), 872 lay in the UTR regions of the protein (6.02%). The splice site mutations reported were 20 (0.14%), whereas 143 (0.99%) were known to be frame shift and 30 (0.20%) as stop gained mutations. Fig 2 depicts the distribution of SNPs under each category.

### Prediction of nature of SNPs

SNPNEXUS and SNAP2 servers were simultaneously used to predict the nature of the SNPs to get high confidence results. A total of 674 nsSNPs were the input for SNPNEXUS, it consults the databases integrated to it and assigns an index score to each reported nsSNP. According to SIFT indexing 298 (44.2%) of the nsSNPs were predicted to be deleterious with the score < = 0.05, out of these 189 nsSNPs were reported to be highly deleterious with a confidence score of 0.00. The PolyPhen indexing which is based on the structural information and Multiple Sequence Alignment categorized 182 of the nsSNPs as ‘probably damaging’ and 112 as ‘possibly damaging’. Hence, 294 (43.6%) of nsSNPs were predicted damaging by PolyPhen.

For the purpose of attaining the highest confident results, 46 nsSNPs were filtered out by combining information from SIFT and PolyPhen. These 46 nsSNPs were then provided to SNAP2 server for further validation. This reported 43 of the nsSNPs to be causing some “effect” on function of protein and only 3 were predicted to be “neutral”. A representation of the results of functional consequences of nsSNPs is in Table 1.

**Table 1:** Impacts of changes in SLC26A4 gene due to amino acid substitution predicted by SNPNEXUS and SNAP2.

dbSNP	Variant	Protein	Mutation	SIFT Score	Prediction	PolyPhen Score	Prediction	SNAP2	Expected Accuracy %
rs111033303	G|T	ENSP00000265715	G209V	0	deleterious	1	probably damaging	effect	95
rs111033380	G|A	ENSP00000265715	G197R	0	deleterious	1	probably damaging	effect	95
rs773076588	T|C	ENSP00000265715	L458P	0	deleterious	1	probably damaging	effect	85
rs758015694	T|C	ENSP00000265715	S427P	0	deleterious	0.999	probably damaging	effect	85
rs529182720	A|C	ENSP00000265715	Q101P	0	deleterious	0.999	probably damaging	effect	91
rs373738509	T|C	ENSP00000265715	W472R	0	deleterious	0.999	probably damaging	effect	91
rs201562855	A|T	ENSP00000265715	N392Y	0	deleterious	1	probably damaging	effect	95
rs776063528	T|A	ENSP00000265715	V359E	0	deleterious	0.999	probably damaging	effect	80
rs147952620	C|T	ENSP00000265715	R409C	0	deleterious	1	probably damaging	effect	95
rs752485540	A|G	ENSP00000265715	Q235R	0	deleterious	1	probably damaging	effect	91
rs111033305	G|C	ENSP00000265715	R409P	0	deleterious	1	probably damaging	effect	95
rs756272252	G|T	ENSP00000265715	G139V	0	deleterious	1	probably damaging	effect	80
rs111033308	G|A	ENSP00000265715	G497S	0	deleterious	1	probably damaging	effect	85
rs121908362	A|G	ENSP00000265715	H723R	0	deleterious	1	probably damaging	effect	85
rs111033344	A|G	ENSP00000394760	D87G	0	deleterious	1	probably damaging	effect	95
rs773173756	T|C	ENSP00000265715	Y127H	0	deleterious	1	probably damaging	effect	85
rs121908360	T|G	ENSP00000265715	F667C	0	deleterious	1	probably damaging	effect	85
rs759264261	G|T	ENSP00000265715	G334A	0	deleterious	1	probably damaging	effect	85
rs111033398	G|A	ENSP00000265715	G95R	0	deleterious	1	probably damaging	effect	95
rs765939287	C|G	ENSP00000265715	S427C	0	deleterious	0.999	probably damaging	effect	57
rs775610413	C|T	ENSP00000265715	R291W	0	deleterious	0.999	probably damaging	effect	95
rs565382433	G|C	ENSP00000265715	Q383H	0	deleterious	1	probably damaging	effect	75
rs111033244	A|G	ENSP00000394760	E384G	0	deleterious	1	probably damaging	effect	95

### Prediction of SNPs associated with disease

The filtered and reduced number of polymorphisms then subjected to the question that whether they are disease associated or not. 46 high confidence nsSNPs were then provided to the SNP&GO and PhD-SNP servers. Both of them calculated a score to declare each nsSNP as neutral or disease associated. The prediction score <0.5 makes the SNP neutral and disease causing if the score is >0.5. Among 46 nsSNPs only one (E303Q) was found to be neutral and the rest 45 nsSNPs were disease causing. A cut off value (p> = 0.80) for PhD-SNP scores was applied to narrow down the number of nsSNPs to 23. Table 2 represents the filtered results.

**Table 2:** Disease association of the SNPs as predicted by SNP & GO and PhD-SN.

dbSNP	Variant	Mutation	PhD-SNP	Prediction	SNP&GO	Prediction
rs111033303	G|T	G209V	0.955	Disease	0.87	Disease
rs111033380	G|A	G197R	0.953	Disease	0.834	Disease
rs773076588	T|C	L458P	0.926	Disease	0.881	Disease
rs758015694	T|C	S427P	0.922	Disease	0.926	Disease
rs529182720	A|C	Q101P	0.911	Disease	0.841	Disease
rs373738509	T|C	W472R	0.904	Disease	0.828	Disease
rs201562855	A|T	N392Y	0.899	Disease	0.87	Disease
rs776063528	T|A	V359E	0.892	Disease	0.911	Disease
rs147952620	C|T	R409C	0.882	Disease	0.95	Disease
rs752485540	A|G	Q235R	0.879	Disease	0.872	Disease
rs111033305	G|C	R409P	0.876	Disease	0.802	Disease
rs756272252	G|T	G139V	0.87	Disease	0.812	Disease
rs111033308	G|A	G497S	0.865	Disease	0.801	Disease
rs121908362	A|G	H723R	0.856	Disease	0.855	Disease
rs111033344	A|G	D87G	0.855	Disease	0.864	Disease
rs773173756	T|C	Y127H	0.846	Disease	0.885	Disease
rs121908360	T|G	F667C	0.839	Disease	0.979	Disease
rs759264261	G|T	G334A	0.839	Disease	0.862	Disease
rs111033398	G|A	G95R	0.836	Disease	0.852	Disease
rs765939287	C|G	S427C	0.807	Disease	0.84	Disease
rs775610413	C|T	R291W	0.805	Disease	0.83	Disease
rs565382433	G|C	Q383H	0.804	Disease	0.81	Disease
rs111033244	A|G	E384G	0.801	Disease	0.80	Disease

### Prediction of effect of stability of protein

I-Mutant and Mu-Pro servers were used to predict the structural effect of 23 candidate nsSNPs, of which only 1 nsSNP (G209V) was predicted by I-Mutant to be increasing the protein stability and rest 22 nsSNPs were suppressing the protein activity by decreasing its stability. Whereas, Mu-Pro predicted none of the nsSNPs to be increasing the protein’s stability. A representation of results is shown in Table 3.

**Table 3:** Effects of SNPs on the Protein Stability, Increasing or Decreasing.

dbSNP	Protein	Mutation	I-Mutant	Mu-Pro
rs111033303	ENSP00000265715	G209V	Increase	Decrease
rs111033380	ENSP00000265715	G197R	Decrease	Decrease
rs773076588	ENSP00000265715	L458P	Decrease	Decrease
rs758015694	ENSP00000265715	S427P	Decrease	Decrease
rs529182720	ENSP00000265715	Q101P	Decrease	Decrease
rs373738509	ENSP00000265715	W472R	Decrease	Decrease
rs201562855	ENSP00000265715	N392Y	Decrease	Decrease
rs776063528	ENSP00000265715	V359E	Decrease	Decrease
rs147952620	ENSP00000265715	R409C	Decrease	Decrease
rs752485540	ENSP00000265715	Q235R	Decrease	Decrease
rs111033305	ENSP00000265715	R409P	Decrease	Decrease
rs756272252	ENSP00000265715	G139V	Decrease	Decrease
rs111033308	ENSP00000265715	G497S	Decrease	Decrease
rs121908362	ENSP00000265715	H723R	Decrease	Decrease
rs111033344	ENSP00000394760	D87G	Decrease	Decrease
rs773173756	ENSP00000265715	Y127H	Decrease	Decrease
rs121908360	ENSP00000265715	F667C	Decrease	Decrease
rs759264261	ENSP00000265715	G334A	Decrease	Decrease
rs111033398	ENSP00000265715	G95R	Decrease	Decrease
rs765939287	ENSP00000265715	S427C	Decrease	Decrease
rs775610413	ENSP00000265715	R291W	Decrease	Decrease
rs565382433	ENSP00000265715	Q383H	Decrease	Decrease
rs111033244	ENSP00000394760	E384G	Decrease	Decrease

### Prediction of PTMs (Post Transcriptional Modifications)

Post Transcriptional Modifications associated with our candidate nsSNPs were predicted by ModPred server by giving the protein sequence as an input. The results were downloaded as .csv format and then analyzed all 23 nsSNPs. ModPred server reported that only 6 nsSNPs (R409C, R409P, D87G, Y127H, G334A, S427C, R291W) located at Proteolytic Cleavage sites and 2 nsSNPs (S427P and S427C) at O-Linked glycosylation sites. The description in given in Table 4.

**Table 4:** Detailed summary of most important SNPs identified.

dbSNP	Mutation	Functional Consequence			Disease Association		Phylogenetics	PTMs
		SIFT	PolyPhen	SNAP2	SNP& GO	PhD-SNP	ConSurf	ModPred
rs111033303	G209V	*	*	*	**	**	9,9,b,s	-
rs111033380	G197R	*	*	*	**	**	9,9,b,s	-
rs773076588	L458P	*	*	*	**	**	7,6,b	-
rs758015694	S427P	*	*	*	**	**	8,8,b	O-linked glycosylation
rs529182720	Q101P	*	*	*	**	**	9,9,e,f	-
rs373738509	W472R	*	*	*	**	**	3,1,e	-
rs201562855	N392Y	*	*	*	**	**	9,9,b,s	-
rs776063528	V359E	*	*	*	**	**	9,9,b,s	-
rs147952620	R409C	*	*	*	**	**	9,9,e,f	Proteolytic cleavage
rs752485540	Q235R	*	*	*	**	**	9,9,e,f	-
rs111033305	R409P	*	*	*	**	**	9,9,e,f	Proteolytic cleavage
rs756272252	G139V	*	*	*	**	**	9,9,b,s	-
rs111033308	G497S	*	*	*	**	**	8,7,b	-
rs121908362	H723R	*	*	*	**	**	8,8,b	-
rs111033344	D87G	*	*	*	**	**	9,9,b,s	Proteolytic cleavage
rs773173756	Y127H	*	*	*	**	**	9,9,b,s	Proteolytic cleavage
rs121908360	F667C	*	*	*	**	**	6,5,b	-
rs759264261	G334A	*	*	*	**	**	9,9,e,f	Proteolytic cleavage
rs111033398	G95R	*	*	*	**	**	9,9,b,s	-
rs765939287	S427C	*	*	*	**	**	8,8,b	O-linked glycosylation
rs775610413	R291W	*	*	*	**	**	9,9,e,f	Proteolytic cleavage
rs565382433	Q383H	*	*	*	**	**	9,9,e,f	-
rs111033244	E384G	*	*	*	**	**	9,9,e,f	-

functional consequences, disease association, PTMs, phylogenetic conservation and minor allele frequency.

(*deleterious

** disease associated, b-buried, e-exposed, f-functional, s-structural).

### Sequence conservation analysis

Any mutation that causes a disease usually resides in highly conserved region of the gene. In order to see the conserved behavior of the 23 nsSNPs, ConSurf server was used. Of the 23 nsSNPs, 8 nsSNPs were highly conserved on the scale along with being structural and buried and 7 were predicted to be highly conserved being functional and exposed.

**Fig 3:**
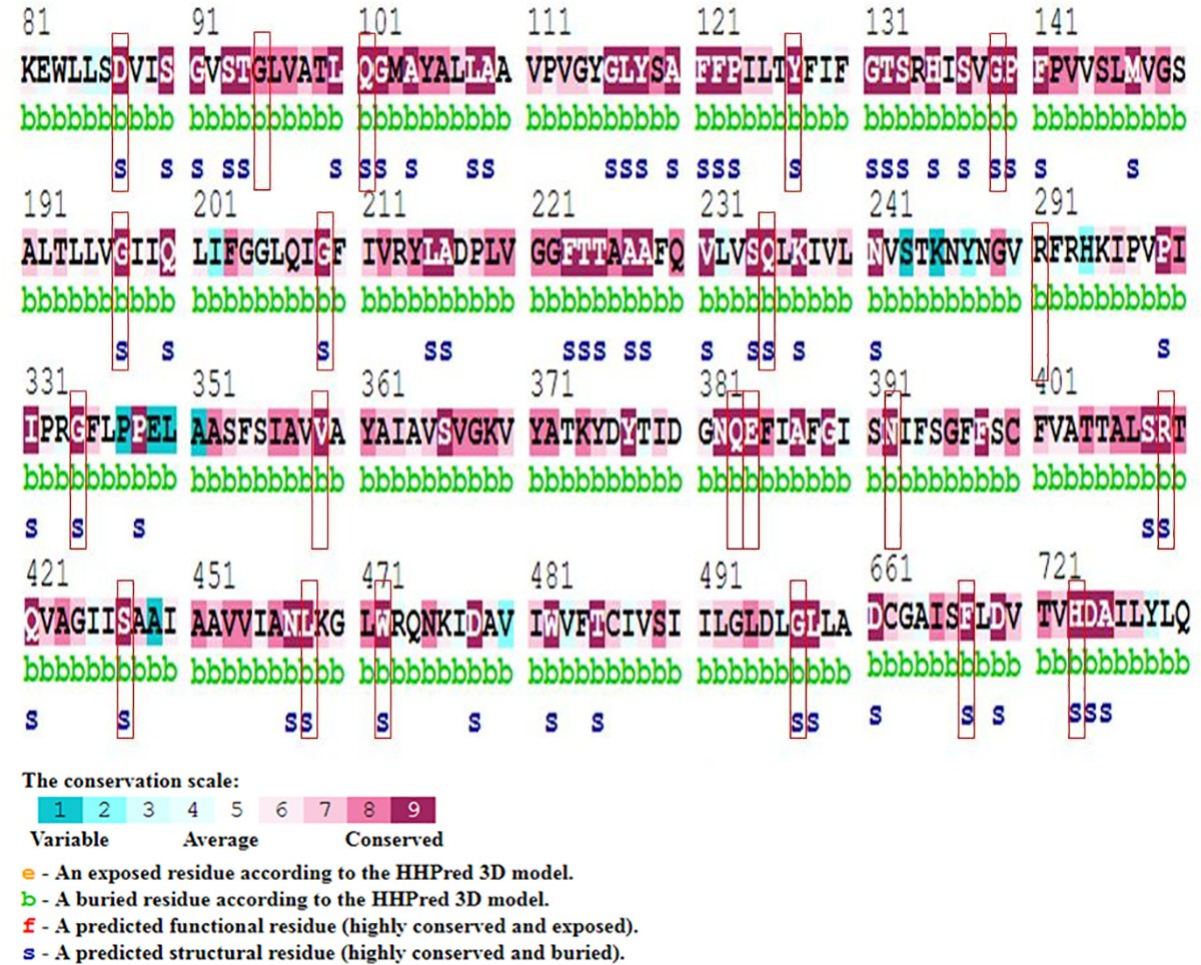
ConSurf results for residues conservation. Colors of ConSurf results showing the level of confidence for the sequence conservation where sky-blue color indicates variables, and dark purple color indicates highly conserved residues.

ConSurf results showed the level of confidence for the sequence conservation in the form of color codes starting from the blue to purple, where blue color indicates the variability and the purple color indicates the highly conserved position. ConSurf predicted the variations to be buried (b) or exposed (e) as well as functional (f) or structural (s) on a range of score 1–9 as depicted by Fig 3. Table 4 depicts a summary for the high confidence 23 nsSNPs including their functional and structural consequences, the PTM sites and Phylogenetic conservation scores.

### Protein modeling

**Fig 4:**
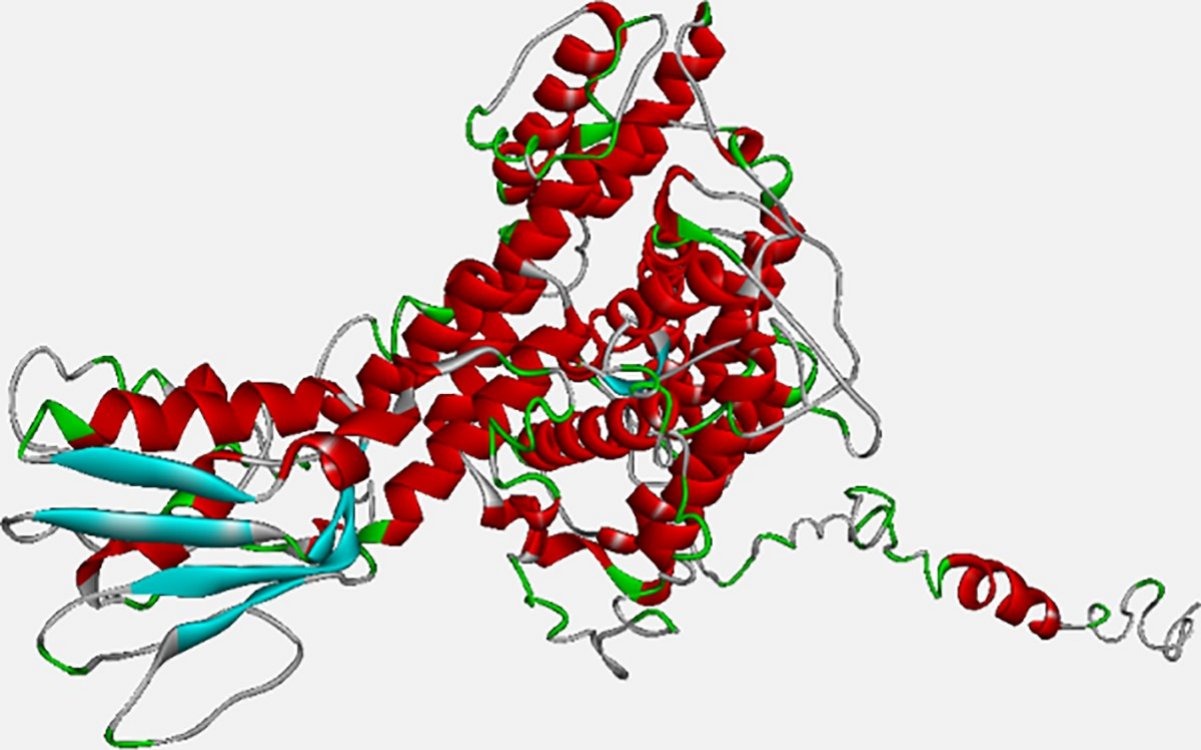
Model structure of SLC26A4. (Pendrin) protein containing 780 a.a. with solid ribbon display style (red, blue and green color: alpha helix, beta sheets and coil, respectively).

**Fig 5:**
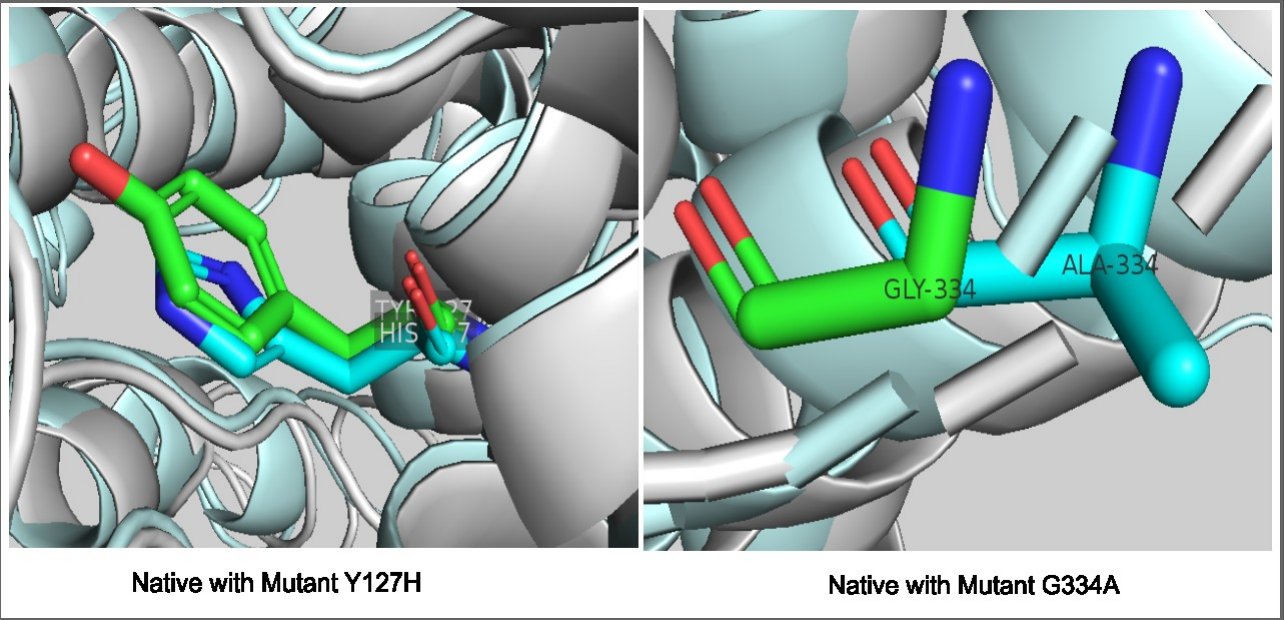
Structure superimposition. Superimposition of native and mutant structures for the two hotspot mutations; native with mutant-1 at point G334A, native with mutant-2 at point Y127H.

Literature revealed that mutations at Y127H and G334A in PDS protein, phenotypically cause diffuse goiter, impaired Thyroid Function Test (TFT) with positive Perchlorate Discharge Test (PDT) and Enlarged Vestibular Aqueduct (EVA). Based on the prediction scores we chose two highly conserved mutations Y127H and G334A to see the protein structural modifications being caused by them. The coiling and recoiling of the predicted structure resulted in the formation of PDS native 3-D structure using threading approaches. By running MODELLER 9.2, we got 7 templates from which 6rtc.1.A and 5da0.1.A showed 38.37% and 37.71% identity with query sequence. We used these templates for the backbone building of our model and structure prediction. The comparative models were generated using Discrete Optimised protein energy (DOPE) assessment score for distinguishing a “good” model from “bad “model. Fig 4 showed the protein structure of PDS native protein. After the prediction of PDS native protein structure via MODELLER, mutant (Y127H and G334A) structures were built using FOLDX. Structure validation done by RAMACHANDRAN plot depicted that 84.5% of the residues of the predicted native structure occupy space in the most favored region, whereas 12.8% occupy space in the additionally favored region. The mutations affect the formation of coiling and angles between folding that cause change in overall structure of proteins. To identify the changes, the mutant structures were superimposed with native structure using structural alignment tool that illustrated the change in formation of 3D domains at point of mutations (Fig 5). The superimposition showed significant change in structure at position Y127H and G334A. The distinct overlapping structures can be seen clearly while the change caused by mutation at position 127 was perceived to be minor. The change in structure also leads to change in functionality that affects the active sites and binding sites of the protein.

### Prediction of binding sites for ligands

Interaction sites present in the protein were firstly identified using COACH along with their binding affinity. For further verification and analysis of the interactions of our native and mutant protein structures with their most suitable ligands, we selected six molecules 4KU, BCT, DMU, IOD, MG, XAN and docked with all the three, native and mutant, structures. The docking interactions were studied with LigPlot that shows the 2D representation of all interactions in particular docking system. From possible binding sites predicted, the positions 622, 674, 704 and 707 carrying amino acids GLU, ARG, GLU and GLY respectively were found to be present in the docking interactions out of which position 674 carrying ARG, position 707 carrying GLY and position 704 carrying GLU were also present in mutants. The interacting residues obtained from docking are presented in Table 5.

**Table 5:** The binding sites predicted at different amino acid residues along with their position and binding affinities.

Position	Amino Acid	Binding Affinity
2	ALA	-0.258
4	PRO	-0.397
320	GLU	-0.307
321	LYS	-0.026
373	THR	-0.039
560	ASP	0.214
564	LYS	-0.044
586	ARG	0.015
590	LYS	-0.114
622	GLU	-0.053
626	GLU	0.058
674	ARG	0.062
704	GLU	-0.392
707	GLY	-0.177

**Fig 6:**
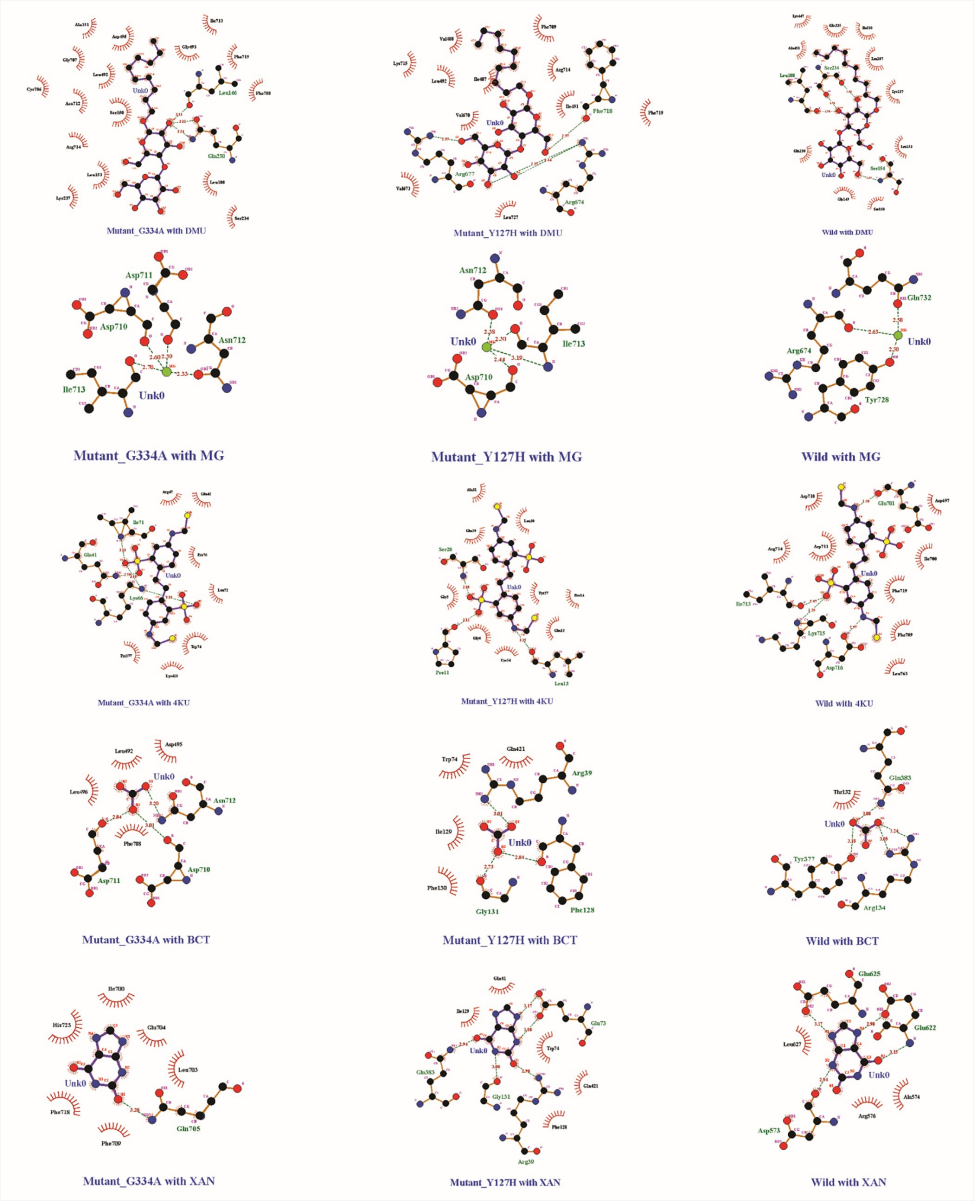
Graphical depiction of protein ligand docking: Native, mutant-Y127H and mutant-G334A protein structures with ligands DMU, MG, 4KU, BCT, XAN show the interactive deviations of mutant1 and mutant 2 from the native structure.

The interactions among ligand and protein residues in mutants were found to be different from the ones in native that indicates the change in functional properties caused by mutations (Fig 6). Here it can be seen that not only the number of interacting residues of protein differs but also the hydrogen bonds and hydrophilic interactions were affected which gives the conformational change of mutation (Table 6).

**Table 6:** Interacting residues obtained from docking. Protein structures Wild, MutantY127H and MutantG334A with ligands 4KU, BCT, DMU, MG and XAN including their binding residues and surrounding hydrophobic interactions.

Receptor—Ligand	Hydrogen Bond Interactions		Hydrophobic Interactions
	Receptor	Ligand	
Wild- 4KU	Glu701, Ile713, Lys715, Asp716	4KU	Asp697, Ile700, Phe719, Leu763, Arg719, Asp711, Asp710
MutantY127H – 4KU	Ser28, Leu13, Pro11	4KU	Gly5, Glu29, Ala31, Leu30, Tyr27, Pro14, Gln12, Cys54, Gly6
MutantG334A – 4KU	Ile71, Gln41, Lys66	4KU	Arg47, Glu42, Pro76, Leu72, Trp74, Lys419, Tyr377.
Wild- BCT	Gln383, Arg134, Tyr377	BCT	Thr132
MutantY127H –BCT	Arg39, Phe128, Gly131	BCT	Phe130, Ile129, Trp74, Gln421
MutantG334A –BCT	Asn712, Asp710, Asp711.	BCT	Phe708, Asp495, Leu492, Leu496.
Wild- DMU	Leu108, Ser234, Ser154	DMU	Ala451, Lys447, Gln235, Ile238, Leu107, Lys237, Leu153, Ser150, Gly149, Gln230
MutantY127H –DMU	Phe719, Arg674, Arg677	DMU	Phe719, Leu727, Val673, Val670, Ile487, Leu492, Lys715, Val488, Phe709, Arg714, Ile491.
MutantG334A –DMU	Leu146, Gln230	DMU	Ile713, Gly493, Phe719, Phe708, Leu108, Ser234, Lys237, Leu153, Arg714, Ser150, Asn712, Cys706, Leu492, Gly707, Ala351, Asp495.
Wild- MG	Gln732, Tyr728, Arg674	MG	-----
MutantY127H –MG	Ile713, Asn712, Asp710	MG	-----
MutantG334A –MG	Asp711, Asp710, Asn712, Ile713	MG	-----
Wild- XAN	Glu625, Glu622, Asp573	XAN	Leu627, Arg576, Ala574
MutantY127H –XAN	Glu73, Gln383, Gly131, Arg39	XAN	Gln41, Ile129, Trp74, Gln421, Phe128
MutantG334A –XAN	Gln705	XAN	Phe709, Phe718, His723, Ile700, Glu704, Leu703.

### Molecular dynamics simulation

#### Temperature, pressure & density

**Fig 7:**
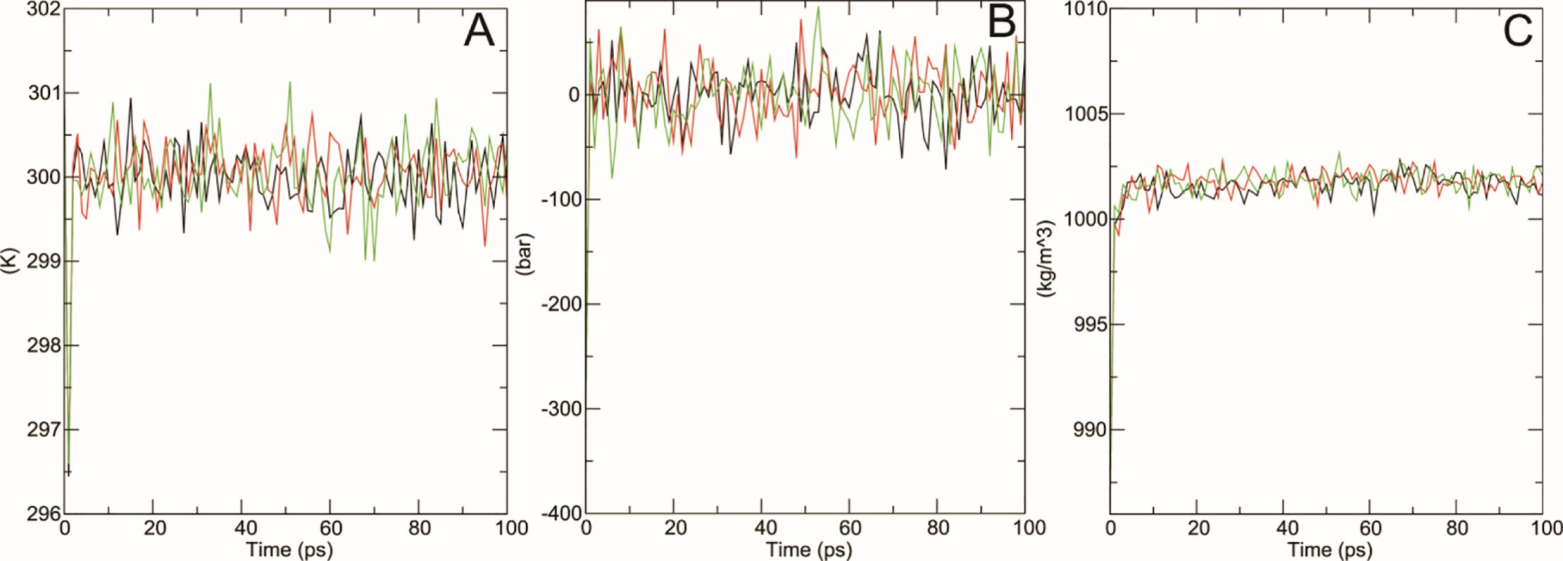
Temperature, pressure and density versus time graph of wildtype and mutants of SLC simulated through GROMACS. Wildtype is shown in blue, G334A mutant is shown in grey and Y127H is shown in orange.

After energy minimization, we equilibrated solvents and ions around our protein structures and in order to establish the proper orientation of molecules in our structures, we performed NVT (thermostat) and NPT (bariostat) equilibration; the two phases to stabilize the temperature and pressure of the systems in isothermal-isobaric ensemble respectively. “gms grompp” tool was used to calculate NVT and NPT of the system. Due to the involvement of temperature, pressure and density in equilibrated process, we established density plot from NPT. Fig 7A showed the temperature plots of native and mutant protein systems at 300K. This plot showed a great fluctuation in temperature for the conformations over the course of 100-ps equilibrium phase. However, this behavior was not much unexpected as the average temperature of most of the conformations was in favorable region where native structure had average temperature of 300.05K while that of mutant Y127H and G334A was 299.97K and 300.74 respectively. Similarly pressure-time graph (Fig 7B) and density-time graph (Fig 7C) were plotted against 100-ps time scale. Pressure-time graph showed that wild type structure had higher peaks above 61.44 bars whereas higher peaks of Y127H and G334A mutant structures were 84.5 bars and 72.3 bars. Local minima in plot for Y127H is -319.5 which was different from the native structure (-309.7 bars) and G334A (-302.1 bars). The pressure-time graph also revealed that the average value of pressure for all conformations for native structure was -0.5 bars but that of Y127H and G334A was -2.56 bars and -1 bar respectively. The average density value of native structure over the course of 100 ps was 1001.4 kgm^-3^ which was very close to the experimental value of 1000 kgm^-3^ while that of mutant Y127H and G334A was more than 1002.6 kgm^-3^.

#### RMSD

**Fig 8:**
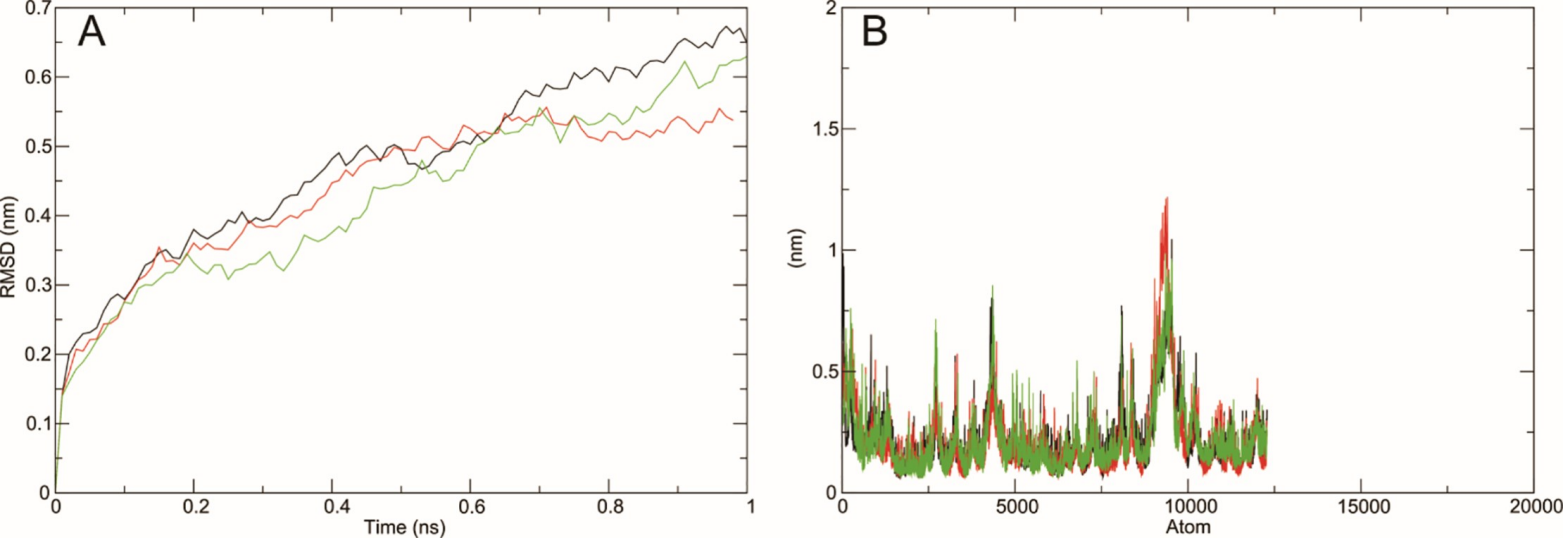
RMSD and RMSF graphs of backbone atoms of wildtype and mutants of SLC simulated through GROMACS version 5.1.2. Wildtype is shown in black, G334A mutant is shown in red and Y127H is shown in green.

Structural and functional behavior of wild type SLC gene and its two mutant G334A and Y127H was compared and visualized using Molecular Dynamics Simulation approach. The main chain root mean square deviations were calculated for the trajectories of native and mutants of RIPK4 protein structures. The RMSD graphs (Fig 8A) were built using the trajectory file for C-alpha backbone least square fit model using g_rms. All the three structures were very close in the starting confirmations and a sudden variation was recorded after 0.15 ns where the RMSD value was 0.15 nm.

Wild type structure attained maximum RMSD value after 3.3 ns whereas the mutants G334A and Y127H attained the maximum values at 3.75 ns and 4.55 ns respectively. The RMSD graph of wild type SLC and its mutants G334A and Y127H that were aligned to the backbone suggested that the wild type SLC gene was steadier than the mutants Y127H and G334A. Secondly these mutants affected the protein dynamics of wild type SLC leading to changes in RMSD graph of backbone of the mutants thus giving an appropriate foundation for further analysis. In order to determine the effect of mutations on dynamic behavior of residues at atomic level, RMSF (Root Mean Square Fluctuation) values of native and mutants were analyzed (Fig 8B). RMSF value of native residues was observed between 0.0602 nm to 1.0602 nm and their average value was 0.2093 nm. The RMSF values of Y127H and G334G residues were observed between 0.0541 to 0.998 with an average of 0.2155 and 0.0561 to 1.2502 with an average of 0.2161 respectively.

#### Radius of gyration and SASA

**Fig 9:**
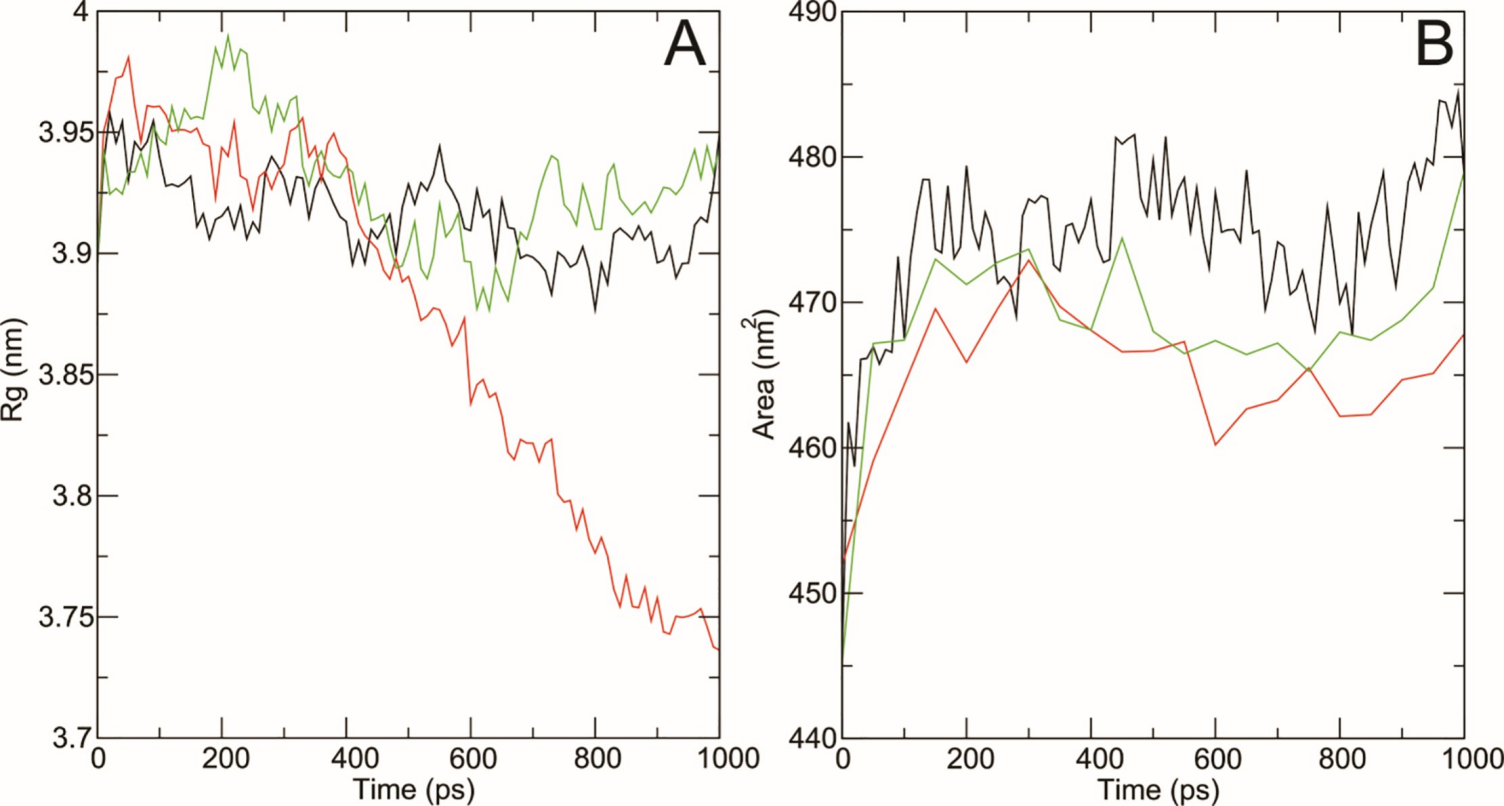
**A) Rg of Cα atoms of native and mutant type SLC protein. B) Solvent accessible surface area of wildtype and mutant type SLC protein** Wildtype is shown in black, G334A mutant is shown in red and Y127H is shown in green.

The radius of gyration (Rg) is defined as root-mean square distance of all atoms from their common center on the basis of mass-weight. It provides an understanding to study the overall dimensions of protein. Radius of gyration plot for alpha carbon atoms (with respect to time) of native and mutant protein structures at 300 K is shown in Fig 9A. At the end of simulation, mutant G334A structure showed a very abnormal behavior with a very low Rg value (< 3.85) for most of the time, whereas native and mutant Y127H structures showed Rg values in a range of 3.85 nm to 3.98 nm.

Solvent accessibility surface area (SASA) is bio-molecular surface area accessibility to solvent molecules. A decrease in the SASA value indicates that the structure is shrunken and the surface area is reduced. Fig 9B showed that the SASA value of native structure was very high ranging from 447 nm^2^ to 484.5 nm^2^ with an average of 473 nm^2^. The SASA value of Y127H and G334A was noted between 445 nm^2^ to 480 nm^2^ with an average of 467 nm^2^ and 453 nm^2^ to 472.5 nm^2^ with an average of 465 nm^2^ respectively.

## Discussion

Pendrin protein like all other proteins of the body holds an important role in the efficient functioning of the human body. It acts as an essential ion exchanger located in many types of cells of the body. Though in the inner ear and thyroid areas, high level of Pendrin expression is known to date. In the areas of expression Pendrin mediates the efflux of Iodide ions across the membranes, which is essential for the secretion or development of hormones including thyroid [36]. Moreover, it has an important role in the acid-base balance, regulation of volume homeostasis and even the mediation of calcium concentration in the endolymph [37]. Any variation, polymorphism, mutation at nucleotide or amino acid level can directly affect the working of the body and thus causing diseases [38].

Among all types of mutations, nsSNPs have the greatest impact on protein structure and function [39]. In Pendrin protein, a total of 14473 SNPs has been reported, and very less work has been done to study the impact of these SNPs, their disease associations, their phylogenetic conservations or their impact on the protein stability or the functional impact. This study aimed at fulfilling this gap, different *in-silico* techniques and tools were used to study the impact and association of the 674 reported nsSNPs. Web based tools including SNPNEXUS, SNAP2, SNP&GO, PhD-SNP, I-Mutant, Mu-Pro, ConSurf and ModPred were used. Each tool was provided with the required input and they helped for extracting useful findings. The methods used in the study basically focus upon the correlation between the variations and their effects on the protein at the molecular level. A variety of tools used for a single purpose make the results confident as each program/tool runs on a different algorithm. Thus, the results obtained from the consensus of all the tools are believed to have the highest accuracy [14].

After the *in-silico* study, 23 nsSNP were found to be most deleterious as per the consensus results of all the tools. The mutations at these particular residues were also found to alter the protein stability. The Post Transcriptional modifications were observed on 8 out of 23 residues. Among deleterious nsSNPs of SLC26A4, two mutations Y127H and G334A were found causing diffuse goiter, impaired TFT with positive PDT and EVA. To examine the impact of these two mutations in the structure of PDS protein (*SLC26A4*) we built protein model of native and mutants and found that there is variation in structure at secondary and tertiary level. Superimposition of mutants with native PDS indicated a significant change in structure at positions G334A and Y127H. To support our conclusion, we carried out protein-ligand docking and Molecular Dynamics simulation of all three structures. The binding poses of the native and mutants with six ligands (4KU, BCT, DMU, IOD, MG, and XAN) were determined and the fitness score of each pose was generated based on the binding affinity of ligand-receptor complexes. The purpose of docking was to see the binding affinity, hydrogen bonds and hydrophobic interactions of the ligands with the protein 3D structures. The results showed that mutation at G334A reduced the hydrogen binding interaction with all six ligands. It was highlighted that, on binding with ligands, the mutation at Y127H and G334A not only affected the binding residues but also the hydrogen bonds and hydrophilic interactions. It has been clearly illustrated that the binding pockets of PDS native protein was accommodating the ligands in a very well manner by making a number of H-bonds and hydrophobic contacts with the active site residues as compared to Y127H and G334A mutants where the residues W500, L323 and R157 were also identified to be present in ligand binding sites. To compare protein dynamics of wild type and mutant SLC, Molecular Dynamics simulation was performed where RMSD, RMSF, SASA, Rg, temperature, pressure and density plots were compared and analyzed at 10 ns MDS.

All these comparisons were performed to observe the conformational behaviors of all structures towards structure stability. After the minimization, we found a little difference between the temperature, pressure and density among native and mutants confirmations over the time. RMSD plots of backbone atoms depicted that wild type structure was much steadier than the mutants. RMSF plots showed that the greatest degree of flexibility was shown by native and mutant structures. The RMSF calculated over the trajectories and averaged over the residual elements showed that the fluctuation of atoms in structures was greater in last quartile. We also examined the radius of gyration and solvent accessibility of surface area analysis to determine that how the unfolding of the mutant protein structure reduces the surface area solubility and how the protein activity is reduced. Higher the Rg value indicates lower the stability [40]. We observed that native structure has less Rg range as compare to the mutants which proved that both mutations Y127H and G334A decrease the stability of protein and hence reduce the protein functionality.

Thus, our study not only presented powerful platform for performing virtual screening of SLC via a consistent and comprehensive procedure, but also revealed the molecular platform for acknowledgment of changes in activity, stability, binding and other properties.

The mutations in the SLC26A4 gene or Pendrin protein are the leading causes for the most common form of Syndromic deafness, Pendred Syndrome [41]. Moreover, little-known role of Pendrin stays in the airway inflammation or hyperactivity, leading to asthma attacks or allergies [42]. The lesser known facts and findings of the study can help understand and interpret the role of these mutations and a step ahead can be taken to cure these diseases or the related ones by other approaches like drug designing etc.

## Conclusion

Current study comprising *in-silico* analysis of nsSNPs of SLC26A4 gene highlighted that 23 nsSNPs (G209V, G197R, L458P, S427P, Q101P, W472R, N392Y, V359E, R409C, Q235R, R409P, G139V, G497S, H723R, D87G, Y127H, F667C, G334A, G95R, S427C, R291W, Q383H and E384G) were the most deleterious among all known and reported SLC26A4 gene nsSNPs. All predicted nsSNPs were both, disease associated and pathological playing significant roles in causing different diseases like deafness, goiter diffusion, and thyroid functional impairment. These nsSNPs were also involved in affecting stability and function of protein. All of them being highly conserved residues also showed post transcriptional modification sites where the residues W500, L323 and R157 were also identified to be present in ligand binding sites.
